# TMS-Evoked Prefrontal Perturbation as a Toy Model of Brain Resilience to Stress During the COVID-19 Pandemic

**DOI:** 10.21203/rs.3.rs-1139350/v1

**Published:** 2021-12-13

**Authors:** Ruben Perellón-Alfonso, María Redondo-Camós, Kilian Abellaneda-Pérez, Gabriele Cattaneo, Selma Delgado-Gallén, Goretti España-Irla, Javier Solana Sánchez, José María Tormos, Alvaro Pascual-Leone, David Bartrés-Faz

**Affiliations:** University of Barcelona; Institut Guttmann; University of Barcelona; Institut Guttmann; Institut Guttmann; Institut Guttmann; Institut Guttmann; Institut Guttmann; Harvard Medical School; University of Barcelona

**Keywords:** TMS, COVID-19, perturbation, brain

## Abstract

Psychosocial hardships associated with the COVID-19 pandemic led many individuals to suffer adverse mental health consequences, however, others show no negative effects. We hypothesized that the electroencephalographic (EEG) response to transcranial magnetic stimulation (TMS) could serve as a toy-model of an individual’s capacity to resist psychological stress, in this case linked to the COVID-19 pandemic. We analyzed data from 74 participants who underwent mental health monitoring and concurrent electroencephalography with transcranial magnetic stimulation of the left dorsolateral prefrontal cortex (L-DLPFC) and left inferior parietal lobule (L-IPL). Within the following 19 months, mental health was reassessed at three time points during lock-down confinement and different phases of de-escalation in Spain. Compared with participants who remained stable, those who experienced increased mental distress showed, months earlier, significantly larger late EEG responses locally after L-DLPFC stimulation (but not globally nor after L-IPL stimulation). This response, together with years of formal education, was significantly predictive of mental health status during the pandemic. These findings reveal that the effect of TMS perturbation offers a predictive toy model of psychosocial stress resilience, as exemplified by the COVID-19 pandemic, and point to the L-DLPFC as a promising target for resilience promotion.

## Introduction

The stressors associated with the coronavirus disease (COVID-19) pandemic, as well as the restrictions imposed to contain the spread of the virus, are increasing the global burden on mental health ^1,2^. The World Health Organization has acknowledged this fact ^3^ and highlighted the importance of integrating mental health into the preparedness and response plans to public health emergencies ^4^. Some studies estimate a 25% increase in the general prevalence of depression and anxiety symptoms ^5^. However, whereas some individuals’ mental wellbeing will be negatively impacted, others - on account of their ‘resilience’ - will not be affected, or even thrive in the face of adversity ^6^.

The concept of resilience is highly heterogeneous with various meanings across different fields of study ^6^. Here we use resilience to refer to the processes that enable an individual to resist the development of illness, mental health problems or distress when confronted with stressful events or trauma ^7,8^. Conversely, psychological vulnerability is reduced ability to cope with stressors, which would constitute a risk factor for developing psychopathology ^9^. Rather than a dichotomy, resilience and vulnerability can be understood as opposite ends of a continuum, which likely reflects the dynamic product of a complex interplay of individual and environmental factors ^10–12^, including genetic and demographic characteristics, socio-economic status, developmental circumstances, access to health care, living conditions, adherence to certain lifestyle factors (e.g. cognitive, physical, nutritional and sleep habits), engagement in emotion-regulation practices such as meditation, social relations and support (particularly early in life), and years of education ^13–17^.

Evidence from animal models and human neuroimaging studies have identified several brain regions and networks that likely play a role in the continuum of resilience and vulnerability. Converging evidence points at the crucial roles of anterior cingulate and insular cortices and their connections within the salience network ^18,19^, as well as limbic structures, such as the amygdala and the ventral striatum ^20^. Additionally, the prefrontal cortex has been identified as an important structure^21^. Specifically, prefrontal cortical volume, activation and connectivity with limbic structures, positively correlate with resilience to traumatic events ^22^; and longitudinal studies using functional magnetic resonance imaging (fMRI) found that children who were better able to regulate amygdala’s emotional response through recruitment of the frontoparietal network exhibited greater resilience to developing depressive symptoms following maltreatment ^23^. Prefrontal function is also involved in the pathophysiology and treatment of psychiatric conditions, most prominently, major depressive disorder ^24^ and schizophrenia ^25^. Finally, the prefrontal cortex appears to not only play a central role in psychological resilience and psychiatric pathophysiology, but has been also proposed as a hub region for cognitive resilience in normal aging ^26^ as well as to brain atrophy and pathology associated with Alzheimer’s disease ^27,28^. Therefore, in the present study we focused on the prefrontal cortex to investigate the neural substrate of resilience to mental health impact of the COVID-19 pandemic and, argued that single pulse transcranial magnetic stimulation (TMS) in combination with electroencephalography (EEG) could be used in human experimental designs akin to the intervention-based animal studies of the neural substrate of resilience.

Animal studies that employ stimuli that can be precisely quantified and controlled, for example using tail-shock stress paradigms ^29^ in which a mild electric shock is given to the tail of rats or mice, illustrate the power of such interventional experimental approaches to gain mechanistic insights into the substrate of resilience. Similar approaches in human research combining non-invasive brain stimulation with neuroimaging are possible. For example, Shafi and colleagues ^30^ have shown that brain responses to TMS allow identifying abnormal cortical activity patterns before the manifestation of clinical symptoms in some forms of epilepsy. More recently, Abellaneda and colleagues ^31^ have shown that the default mode network’s response profile to intermittent theta burst stimulation of the inferior parietal lobule can be used to predict cognitive decline or maintenance after a three-year follow-up in an aging population, well over and above of what baseline neuroimaging data alone could predict. Furthermore, recent methodological advances ^32,33^, have revealed that single pulse TMS can be used concurrently with EEG to produce highly specific and reliable cortical response profiles.

**Figure 1:**
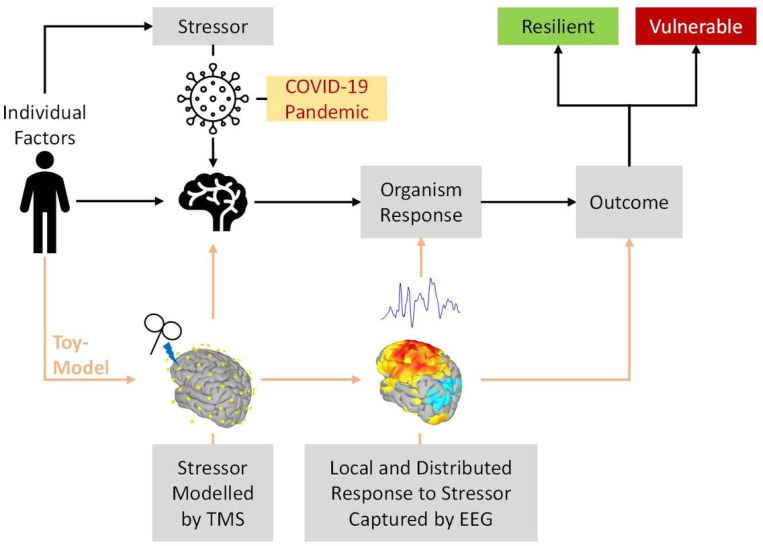
Schematic Illustration of the toy-model approach in the study design. MRI-guided single pulse TMS perturbation is used to experimentally model as the stressor, captured by evoked EEG reactivity, and measured at local and global levels. We examined whether this ‘toy-model’ can predict the eventual impact of the COVID-19 on mental health assessed months later. We further hypothesized the stressor would be moderated by demographic and individual factors such as years of formal education. Modified from Pascual-Leone and Bartrés-Faz 6.

We propose that TMS-EEG can be a ‘toy model’ of the impact of a stressor onto an individual brain and provide a quantitative observation of the effect of the controlled external perturbation on brain dynamics that can be used to test specific predictions about a complex system. In theoretical physics ‘toy-models’ refer to simple models which nevertheless provide a quantitative explanation and reliable prediction of a given phenomenon ^34,35^. Specifically, as illustrated in Fig. 1, here we use the EEG response to TMS as a ‘toy model’ predictive of the eventual (months later) impact of the COVID pandemic and confinement, on potential development of mental health problems. We hypothesized that individual differences in the cortical response to TMS brain perturbation of the left dorsolateral prefrontal cortex, compared to another control cortical target (i.e., inferior parietal lobule), and recorded using EEG, would be predictive of psychological distress outcomes during the COVID-19 pandemic and confinement. Our findings contribute to the understanding of biological brain mechanisms of resilience, and identify a potential target and novel strategy to promote individual resilience to unexpected stressors.

## Results

### The dynamics of the EEG response to TMS perturbation differentiate individuals eventually found to be ‘resilient’ from those ‘vulnerable’ to the mental health impact of the COVID-19 pandemic

Assessments of mental health using the four-item patient health questionnaire (PHQ-4), an ultra-brief depression and anxiety screening self-report questionnaire, were obtained prior to the COVID-19 pandemic and up to three additional times during the pandemic. If during all timepoints across the confinement, the PHQ-4 score was lower or equal than before the pandemic outbreak, subjects were classified as resilient (n=32). Conversely, if a given subject had a higher score at any time point during the pandemic, they were classified as vulnerable (n=32). Because not all participants completed stimulation at both target locations, the subgroups used in this analysis were smaller for each target (for L-DLPFC, 23 resilient and 25 vulnerable; for L-IPL, 22 resilient and 23 vulnerable). To make sure that the mental health impact of the pandemic was related to the levels of stress perceived during the outbreak, we correlated the average score of the three pandemic PHQ-4 timepoints, with the scores of the 14-item perceived stress scale ^36^, which was also completed by participants during the pandemic, and found a strong positive correlation (*R*_*s*_=.69; *p*<.001), indicating that subjects experiencing more stress during the pandemic also had a larger mental health impact. Overall, participants had a low to moderate level of perceived stress during the pandemic (*Mdn*=14; range from 2 to 32).

**Figure 2:**
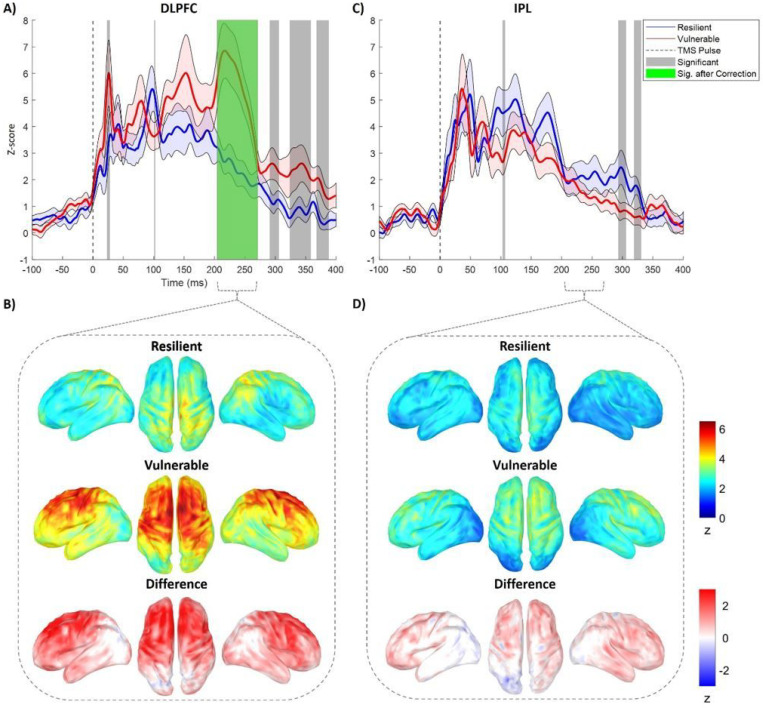
Results of permutation testing of the difference between resilient and vulnerable individuals on the TMS evoked EEG time-series. A) shows the significant differences at the local EEG time-series during L-DLPFC stimulation in grey horizontal bands, while the horizontal green band highlights the cluster surviving correction for multiple comparisons. B) shows the topographical distribution in source space of the response to L-DLPFC stimulation during the green shaded time-window in A, for both groups and their difference. C) depicts the results of permutation testing for the control stimulation of the L-IPL. D) shows the topographical distribution for the same time window as B, for both groups and their difference. Red and blue contours along the plot lines in A and C depict the standard error of the mean. DLPFC, dorsolateral prefrontal cortex; IPL, inferior parietal lobule

Point-by-point non-parametric permutation testing (1000 permutations) with cluster correction for multiple comparisons ^37^ on the TMS-EEG evoked time-series, revealed a single broad cluster (i.e., 202–269 ms post-stimulus) surviving correction for multiple comparisons, only during stimulation of the left dorsolateral prefrontal cortex (L-DLPFC; see Figure 2, A). Inspection of the topographical distributions in source space for the surviving cluster, confirms that vulnerable individuals had a qualitatively stronger frontal activation than resilient ones (see Figure 2, B). There were no significant clusters revealed after analysis of the responses to the left inferior parietal lobule (L-IPL) control target (see Figure 2, C and D), nor for the distributed responses to stimulation on either target (Figure S1).

### Local EEG response to TMS perturbation of the left dorsolateral prefrontal cortex predicts mental health during the pandemic’s lockdown confinement

Without classifying participants into resilient or vulnerable, a multiple linear regression model was fit to determine the potential of TMS evoked EEG perturbation of the L-DLPFC to predict mental health outcomes after the COVID-19 pandemic outbreak and the strict lock-down confinement imposed to curb community transmission of the virus. The model’s response variable was the mean of the total scores for the three PHQ-4 questionnaires, which were completed by participants during the lock-down confinement. Candidate predictors were the local and global brain EEG reactivity to the TMS pulse — recorded before the pandemic outbreak —, as well as their interaction with the stimulation target definition method (i.e., functional or anatomical). Additionally, we included age, gender, and years of formal education as predictors, because these are demographic and individual factors partially predictive of resilience to stress ^17^. Finally, we included as a predictor the number of months before the pandemic since each subject underwent TMS-EEG. This was included to control for the possibility that the amount of time passed from stimulation to pandemic would have an impact in the prediction.

**Figure 3:**
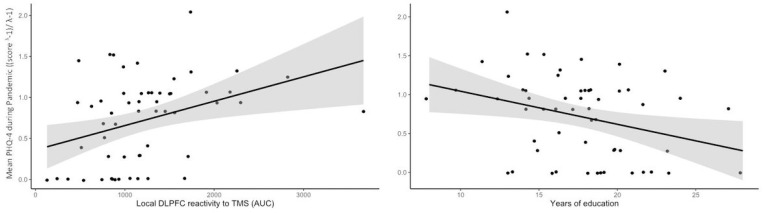
Scatter plots illustrating the linear relationship between the predictors and the response variable in the reduced model. Black line in each plot depicts the least squares regression line; shaded grey contours depict 95% confidence intervals. The response variable depicted here in the y-axis is Box-Cox transformed (λ=−0.078). PHQ-4, four item patient health questionnaire; DLPFC, dorsolateral prefrontal cortex; AUC, area under the curve.

The full linear regression model for the L-DLPFC stimulation target significantly predicted mental health during the pandemic (*F*(8,47)=3.1, *p*=.006, *R*^*2*^_*adj*_=.243), and revealed as significant predictors the local brain reactivity to TMS (*t*=2.31, *p*=.025) and years of formal education (*t*=−2.98, *p*=.005). See supplementary table S1 (model “Full DLPFC”) for detailed results. Both predictors were independent from each other, as reveled by the lack of correlation between them (*R*^*2*^=.187, *p*=.167). Subsequently, we tested a reduced model (*F*(2,53)=10.5, *p*<.001, *R*^*2*^_*adj*_=.257) retaining only as predictors local brain reactivity (*t*=2.33, *p*<.024) and education (*t*=−2.86, *p*=.006). See supplementary table S1 (model “Reduced DLPFC”) for detailed results. Likelihood ratio test comparing the two models showed that the full model did not provide a better fit than the reduced one (*χ*^*2*^(6)=4.98, *p*=.546). The lower Akaik and Bayesian information criteria (AIC and BIC, respectively) values for the reduced model further suggest a better and more parsimonious fit (AIC_full_=79.83, AIC_reduced_=72.82; BIC_full_=98.06, BIC_reduced_=78.89). Fig. 3 illustrates the linear relationship between the significant predictors and the response variable in the reduced model. Analysis of variance of the reduced model revealed that local L-DLPFC reactivity explained 12.79% of total variance in mental health during the pandemic, while education explained 15.64%.

To assess the brain specificity of our findings, we fitted a model replacing the predictors for local and global EEG reactivity with those measured when stimulating the L-IPL. The regression model for this control stimulation target did not significantly predict mental health during the pandemic (*F*(8,46)=0.4, *p*=.915, *R*^*2*^_*adj*_=−.097). See supplementary table S1 (model “Full IPL”) for detailed results.

Finally, to demonstrate the specificity of the stimulation itself, a model was fitted where we added the local baseline pre-TMS activity as an additional predictor to the reduced L-DLPFC model. The resulting model, while still significant (*F*(3,47)=7.09, *p*<.001, *R*^*2*^_*adj*_=.25), revealed that baseline pre-TMS EEG activity did not significantly contribute to predict mental health during the pandemic (*t*=.67, *p*=.507). See supplementary table S1 (model “Reduced+Baseline”) for detailed results.

## Discussion

We tested the EEG brain reactivity to TMS perturbation as a toy model of resilience in the face of the COVID-19 pandemic and lockdown confinement. The results show that the local response to TMS perturbation of the left DLPFC — measured months *before* the pandemic outbreak — offers a predictive marker of the future mental health impact of the pandemic and confinement. These results serve as a proof of concept that the toy-model using the TMS pulse as an external transitory insult allows quantification of the brain responses and identifies critical and specific substrates of resilience or vulnerability to more complex stressors. At the core of this ‘toy-model of resilience’ is the assumption that TMS stimuli can be understood as stressors or insults in themselves. This is supported by evidence showing that the stimuli interfere with ongoing brain activity by suddenly injecting an amount of current into the neural circuitry, which then results in phase resetting and TMS-evoked perturbation of the EEG ^38^. Indeed, failure to suppress this perturbation can lead — in the presence of pathological conditions such as stroke or epilepsy — to a cascading synchronization of neuronal activity that in turn might lead to a seizure ^39,40^. Thus, we can interpret the present results as showing that in the presence of such a brain state disruption, a more resilient brain is better able to tolerate the perturbation. A link between the ability of the brain to withstand a targeted attack and resilience, has also been proposed by Santarnecchi and colleagues employing in-silico models ^41^, but the present study is the first to offer direct experimental support on a topic of substantial timely relevance.

Our findings are specific to TMS perturbation of the L-DLPFC, because neither the response to L-IPL stimulation, nor the pre-TMS baseline EEG activity held significant predictive value. Moreover, we show that the findings are restricted locally to the stimulated area, because the distributed measure of response did not yield significant predictive value. Nevertheless, years of formal education was also found to be predictive of mental health during the pandemic, which is unsurprising given the well-known epidemiological-level notion that individuals with higher socioeconomic status (encompassing, among others, educational attainment and income) have lower odds of being depressed ^42^, and that education might be a protective factor both against cognitive as well as emotional vulnerability, by boosting a higher efficiency on top-down emotional regulatory processes ^43^. In this context, and while education and TMS-EEG reactivity were independent from one another in this analysis, it is still plausible that both reflect prefrontal function.

Dividing the sample into resilient and vulnerable participants, based on the changes in mental health during the pandemic, allowed us to directly compare the dynamics of the EEG response to the TMS perturbation, revealing that the most discriminative time segment after TMS perturbation of the local L-DLPFC is the late TMS evoked response between 202ms and 269ms post-TMS. Interestingly, this occurs in the vicinity of the commonly found P180 EEG evoked component in response to single TMS pulses of the primary motor cortex ^44^. This component has been found to significantly decrease after application of voltage-gated sodium channel blockers, such as lamotrigine and carbamazepine ^45,46^, indicating that this component reflects cortical excitability. Therefore, we propose that higher amplitudes found in vulnerable individuals may be reflective of cortical hyperexcitability. Furthermore, the amplitude of late TMS-EEG responses (≥180ms) is related to GABA-B mediated inhibition, as it is significantly reduced after long interval intracortical inhibition ^47^. Therefore, the increased amplitude in late EEG responses found in vulnerable individuals when compared to resilient ones might reveal a relatively lower intracortical inhibitory capacity and point to differential levels of activation of parvo-albumin positive cells and integrity of peri-neural nets — main substrates of intracortical excitability-inhibition balance ^48,49^. This would predict that conditions that alter and disrupt parvo-albumin positive cells and integrity of peri-neural nets, such as status post traumatic brain injury, early stages of Alzheimer’s disease, or schizophrenia, would be associated with a loss of resilience and increased vulnerability to stressors. Epidemiologic data appear to support such notions ^50–52^ .

Our results are novel and relevant in advancing our understanding of the neural mechanisms of resilience, however, this study has some limitations. We had to conduct the analysis on each stimulation target separately due to missing data, which may have hindered statistical power. The regression analysis results would benefit from further validation on a separate independent sample to be able to make reliable predictions of mental health outcomes based on the response to TMS perturbation. Moreover, the changes in mental health observed during the pandemic were small overall, with most participants not surpassing clinical screening thresholds for depression and anxiety, therefore a sample with a broader range of mental health impact could provide a clearer picture of the neurophysiological determinants of such impact. However, despite the narrow range of mental health changes, we are still able to show that TMS-EEG can detect a neurophysiological signature underlying the future differential impact of the pandemic on mental health.

The presented results are not only relevant as a proof of concept for using intervention-based designs in neuroimaging investigations of the neural basis of resilience, but also add to the existing evidence of a primary role of the left prefrontal cortex in resilience processes ^20,22,23,26,26–28,53−55^. This, in turn, singles out the DLPFC as a promising target for interventions aiming to promote resilience, including the potentially transformative possibility of using non-invasive stimulation to promote brain resilience by modulating prefrontal brain activity. Several of our results and other lines of evidence support such potentially transformative therapeutic intervention: (1) the known protective role of higher prefrontal function to the deleterious mental health effects of stress and trauma ^22,23^; (2) the link we have demonstrated between the L-DLPFC response to an insult and the resilience capacity when facing the stressors associated with the pandemic; (3) our finding of exaggerated response to TMS perturbation of the L-DLPFC in individuals that would be vulnerable to pandemic related stress; and (4) the established ability of non-invasive stimulation techniques to induce long lasting brain plastic changes ^56^. Finally, the identified resilience signature in the local DLPFC response to TMS perturbation is a potential neurophysiological marker of resilience capacity that might be useful in a preventive precision medicine framework, when assessing the potential risk of deleterious mental health impacts for a given individual, when exposed to future stressful events such as a new pandemic.

## Methods

### Study design

**Figure 4:**
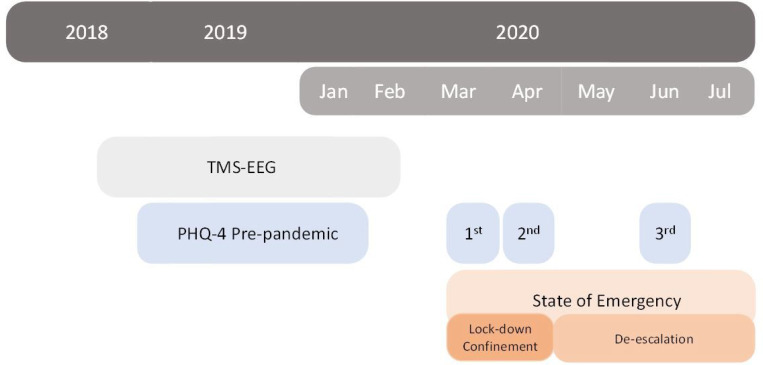
Timeline of relevant events for the study cohort. According to the Spanish Government state of alarm dictation orders and subsequent de-escalation (https://www.boe.es/eli/es/rd/2020/03/14/463/con). 1st, 2nd and 3rd indicate the three PHQ-4 based mental health monitoring timepoints during the pandemic. PHQ-4, four item patient health questionnaire; COVID-19, coronavirus diesease 2019; TMS-EEG, transcraneal magnetic stimulation with concurrent electroencephalography.

In the present study we analyzed existing data from participants of the longitudinal study ‘Barcelona Brain Health Initiative’, BBHI for short ^57^. In mid-March 2020, during the COVID-19 epidemic, the BBHI launched a longitudinal substudy to investigate the mental and brain health impact of societal and personal restrictions imposed by the pandemic ^58,59^. For the present report, we selected those BBHI participants who had undergone concurrent TMS-EEG between July 2018 and February 2020, before the COVID-19 pandemic outbreak, as well as mental health monitoring before and during the lockdown using the Patient Health Questionnaire for Depression and Anxiety (PHQ-4), a standardized ultra-brief tool for detecting both anxiety and depressive disorders ^60^. The scale was administered at four different time points; one between November 2018 and January 2020, hence before a mandatory lockdown that was issued by the Spanish Government on March 14th 2020, and another three timepoints during the pandemic, spanning a total of 3 months during the strictest home-confinement and initial phases of de-escalation (see Figure 4).

The sample included 74 healthy adults (34 females) ranging from 42 to 66 years (*M*=55.07; *SD*=7.1), with a range of years of formal education from 8 to 28 years (*M*=18.01; *SD*=3.85). Consistent with the BBHI general inclusion criteria, none of these individuals reported a medical diagnosis of any major neuropsychiatric disorder (including mood and anxiety disorders) at study entrance and had normal cognitive function as assessed by comprehensive neuropsychological testing ^57^. All participants gave written informed consent, and the local ethics committee (Comité d’Ètica i Investigació Clínica de la Unió Catalana d’Hospitals) approved the protocols here described and conformed to the Declaration of Helsinki for research involving human subjects.

The objective of this analysis was to evaluate the potential of using the brain response to TMS perturbation – quantified by EEG – as a toy model of psychological resilience to a complex stressor, namely, the COVID-19 pandemic and confinement, the impact of which was quantified with the PHQ-4 questionnaires. Given the known involvement of the prefrontal cortex in various forms of resilience, we hypothesized that the EEG response to left dorsolateral prefrontal stimulation would be predictive of mental health during the pandemic. Stimulation on the left inferior parietal lobule was included in the analysis as a control stimulation condition.

### Neuronavigated TMS-EEG

Transcranial magnetic stimulation was delivered over the left dorsolateral prefrontal cortex (L-DLPFC) and the left inferior parietal lobule (L-IPL). Stimulation was guided by a BrainSight neuronavigation system (RogueResearch, Inc., Canada). Targets were determined for each individual based on either anatomy or the cortical parcellation by Yeo and colleagues ^61^. See supplementary materials for MRI acquisition parameters and target determination procedures. Stimulation intensity was 120% of resting motor threshold, determined as the minimum intensity required to elicit motor evoked potentials in the first dorsal interosseous muscle of the relaxed right hand, of at least 50μV peak-to-peak, in at least five out of ten trials ^62^. For each target, 120 single biphasic pulses were delivered through an MCF-B65 butterfly coil, using a MagPro X100 stimulator (Magventure, Inc., Denmark). The order of targets was randomized for each participant. Stimulation was performed concurrently with EEG using a TMS compatible ActiChamp 64-channel system with active electrodes (BrainProducts, GmbH., Germany). Electrode impedance was monitored and kept under 5kΩ throughout the experiment. EEG data was recorded DC to 500Hz and digitized at a 1KHz sampling rate. While 76% of participants completed stimulation of the L-DLPFC and 74% completed stimulation of the L-IPL, only 50% of participants completed stimulation on both targets. For this reason, statistical analysis was conducted separately for each stimulation target.

### Mental health assessment

Mental health was measured using the PHQ-4, an ultra-brief four item depression and anxiety screening self-report questionnaire, that consists of a 2-item depression scale (PHQ-2) and a 2-item anxiety scale (GAD-2). Each subitem scores in the range of 0 to 6, with combined range from 0 to 12. On each subscale a score of 3 or greater is considered positive for screening purposes. The test was administered a total of four times in an online format, once before the pandemic and at three timepoints during the confinement and de-escalation. All participants in this analysis completed the pre-pandemic questionnaire and most completed the three additional ones during the pandemic (69%), however, few participants completed only two (23%) or one (8%) of them. For the purposes of quantifying mental health status during the pandemic in the regression models, we used the mean of total scores from the completed questionnaires during the pandemic.

### EEG data preprocessing and analysis

All EEG data was preprocessed using functions from the EEGLAB toolbox ^63^ and the TESA plugin ^64,65^, as well as custom made Matlab (The MathWorks, Inc., USA) scripts. Source reconstruction and analysis was performed using Brainstorm ^66^ and custom made Matlab scripts.

First, the data was segmented around the TMS pulse (−1000 to +2000 ms from the pulse) and baseline corrected (−900ms to −100ms from the pulse). Then the direct electrical pulse artifact (between −2ms and 14ms from pulse) was zero-padded. Bad channels were then identified via visual inspection and removed (range from 0 to 3; *M*=0.49, *SD*=0.76). Bad epochs were first tagged based on threshold voltage (>100 μV), probability and kurtosis using the inbuilt TESA plugin functions, visual inspection ensured that the epochs were correctly tagged and that no bad epochs were missed, then they were removed from further analysis (range from 0 to 53; *M*=20.59; *SD*=9.66). A first round of fast independent component analysis (ICA) was used to reject any remains of the immediate electrical pulse artifact (range from 0 to 3; *M*=0.67; *SD*=0.65). The zero-padded pulse artifact was then linearly interpolated, and the data was re-referenced to the average of all channels. Finally, a second round of ICA was used to reject any other remaining artifacts (e.g., muscle, eye-movements, heartbeat and others), as well as the somatosensory and auditory potentials evoked by transcutaneous scalp nerve excitation and coil firing sounds, respectively (range from 21 to 23: *M*=28.37; *SD*=2.77). These are commonplace preprocessing procedures for TMS-EEG data and have been described in greater detail elsewhere ^65^.

The cleaned preprocessed data was then used for source reconstruction in Brainstorm. For each subject a forward model was estimated via the openMEEG algorithm ^67^ using each subject’s T1 and T2 weighted MRI images and digitized real electrode locations, when available (the 29 subjects for which anatomical target determination was used, had no digitized electrode locations, therefore, the standard 10–10 electrode locations were used instead). The inverse solution was estimated using the minimum norm imaging method ^68^. Sources were then computed as current density maps for constrained orientations only (i.e., normal to cortex). These are commonplace source reconstruction procedures for TMS-EEG data and have been described in greater detail elsewhere ^32,33^.

### Local and distributed EEG measures of the response to TMS perturbation

To quantify EEG derived brain reactivity measures to the TMS pulse we computed the following global (i.e., distributed) and local reactivity measures:

**Global response:** the global mean field amplitude ^69^ of the TMS evoked potentials (TEPs), was taken as the estimated time-series of the response to the TMS pulse.

**Local response:** To extract local measures, we first defined a region of interest (ROI) of 100 vertices around each subjectś stimulation target coordinate, which corresponds to a cortex surface area of approximately 10 cm^2^. Then the TEP response at the targeted location in source space was taken as the local reactivity time-series. To allow group level statistics, the TEP time-series in source space of each vertex within the target ROI were rectified, averaged together, and then normalized via z-score transformation

z=(TEP-μ)/σ

Where μ is the average of the pre-stimulus baseline (−500ms to −3ms) and σ is the standard deviation of the baseline.

For both global and local TMS response measures, the trapezoidal integration from 15 ms to 400 ms post-TMS stimulus was used in the regression analyses as overall response to TMS perturbation.

### Statistical analysis

Statistical analysis was performed in RStudio ^70^ and Matlab 2020b.

To compare the global and local TMS evoked time-series for each target we conducted four permutation-based tests. In each test, we computed the difference of means for each data point within the time-series time-window of interest (from 15 ms to 400 ms after the TMS pulse). In each of the 1000 permutations, the labels for each group (resilient or vulnerable) were scrambled. The resulting p-values were adjusted for multiple comparisons using cluster correction ^37^, whereby the size and magnitude of a given cluster of significant timepoints is considered to survive correction if the size and magnitude of the cluster is above 95% of all cluster sizes and magnitudes discovered during permutation testing. To test the correlation between perceived stress and mental health during the pandemic a Spearman rank correlation was used.

To investigate the predictive value of TMS reactivity at a local and global levels we used two multiple linear regression models (i.e., one for each stimulation target). The full model included as predictors the local and global TMS brain reactivity measures, age, gender, and years of education. Additionally, and to control for the possibility that the target definition method influenced the candidate reactivity measures, we included the interaction between the targeting method (2 levels: anatomical or functional) and both local and global reactivity measures. In each regression model the response variable was each subject’s mean of the completed pandemic PHQ-4 scores. In the presence of significant predictors, a reduced model including only those was defined and compared against the full model. To determine the better fitting model, we used the likelihood ratio test and further confirmed the result based on the AIC and BIC values. Finally, to assert the TMS induced specificity of the findings, we fitted an additional model where the possible contribution of the pre-TMS stimulus baseline to the prediction of mental health was tested.

Lilliefors test on each model’s residuals revealed that they were not normally distributed, therefore, we transformed the response variable in each model using Box-Cox transformation, resulting in normally distributed residuals, therefore, the results reported in this work correspond to the regression models with the transformed response variable. Assumptions of multicollinearity, autocorrelation and heteroscedasticity were met in each model.

## Supplementary Material

Supplement 1
