# Supplementary material for: TMS-Evoked Prefrontal Perturbation as a Toy Model of Brain Resilience to Stress During the COVID-19 Pandemic
Source: Res Sq. 2021 Dec 13:rs.3.rs-1139350. Preprint. [Version 1] doi: 10.21203/rs.3.rs-1139350/v1 (PMC8687479; doi:10.21203/rs.3.rs-1139350/v1)
Supplement: Supplement 1 [file 5416c23a6f70aa2f31dfc132.docx]

Supplementary Materials for

TMS-evoked prefrontal perturbation as a toy model of brain resilience to stress during the COVID-19 pandemic

Ruben Perellón-Alfonso, María Redondo-Camós, Kilian Abellaneda-Pérez, Gabriele Cattaneo, Selma Delgado-Gallén, Goretti España-Irla, Javier Solana Sánchez, José M. Tormos, Alvaro Pascual-Leone, David Bartrés-Faz

Correspondence to: DBF, [dbartres@ub.edu](mailto:dbartres@ub.edu); APL, apleone@hsl.harvard.edu

**This file includes:**

Structural MRI acquisition parameters

TMS target determination procedures

Figs. S1 and S2

**References**

Table S1

Structural MRI acquisition parameters

T1 and T2-weighted anatomical MRI scans were obtained for all participants and used for neuronavigation and EEG source reconstruction. Participants undertook a high resolution (0.8x0.8x0.8mm^3) 3D MP-RAGE T1 weighted structural magnetic resonance image obtained from a 3T Siemens Magnetom Prisma machine. A total of 208 contiguous axial slices were obtained in ascending fashion (sequence parameters of repetition time = 2400ms, echo time = 2.22ms, TI = 1000ms, flip angle = 8º, slice thickness=0.8 mm and field of view =256mm). Additionally, a high resolution (0.8x0.8x0.8mm^3) 3D SPC T2 weighted structural magnetic resonance image was obtained from the same machine (sequence parameters of repetition time = 3200ms, echo time = 563ms, flip angle = 120º, slice thickness=0.8 mm and field of view =256mm). Image quality control measures were implemented manually by a trained MRI technician to ensure that these did not contain MRI artifacts or excessive motion. The T1 was used for neuronavigation, while the T2 was used, together with the T1, to produce high quality segmentations and meshes for EEG source reconstruction.

TMS target determination procedures

For the 29 first subjects of the sample, which were recorded in 2018, individualized targets were determined anatomically. Left-DLPFC stimulation was targeted at the superior half of the middle frontal gyrus, approximately 3 cm anterior to the precentral sulcus. Left-IPL was targeted at the superior edge of the angular gyrus, roughly 1cm inferior to the intraparietal sulcus. For the remaining 45 subjects, which were recorded between 2019 and 2020, targets were determined based on the group-level resting-state seven functional networks parcellation by Yeo and colleagues ^1^, according to the method described first by Ozdemir and colleagues ^2^. Briefly, confidence maps for each resting state network across a sample of 1000 healthy subjects were used. In these maps, each vertex has a confidence value of belonging to a particular network (ranging from -1 to 1), with larger values indicating higher confidence. Using these maps at the group level it is possible to select the most consistent and reliable regions, within the angular gyrus and the middle frontal gyrus. Each individual’s T1 was then linearly transformed to the MNI space. Finally, the invers transform was used to return the coordinates of interest to each subject’s native space, by using the FSL’s ^3^ FNIRT tool. These individual coordinates were then used to guide stimulation using a BrainSight neuronavigation system (RogueResearch, Inc., Canada).

The mean MNI coordinates of the anatomical targeting method were *x*=-44, *y*=-67, *z*=44 for IPL and *x*=-33, *y*=37, *z*=48 for L-DLPFC. While mean coordinates of the functional targeting method were *x*=-53, *y*=-51, *z*=18 for IPL and *x*=-43, *y*=34, *z*=42 for L-DLPFC. Figure S1 shows these mean target coordinates on the MNI template.

In order to account for a possible effect of the targeting method on the predictors of interest, target determination method was included in the main analysis as an interaction term for each TMS-EEG reactivity measure.

**Fig. S1.** Mean L-IPL and L-DLPFC coordinates for each targeting method overlayed on the MNI template. Yellow and red ROIs correspond to a projection of the PFC and IPL, respectively, from the 17- network Schaefer parcellation of the Yeo atlas ^4^.

**Fig. S2.** Uncorrected significant results after permutation testing of resilient against vulnerable individuals for global reactivity to DLPFC and IPL stimulation. Grey regions highlight significant differences between curves prior to cluster correction for multiple comparisons. Shaded blue and red contours along the curves depict the standard error of the mean. GMFA: global mean field amplitude. DLPFC, dorsolateral prefrontal cortex; IPL, inferior parietal lobule.

**References**

1. Thomas Yeo, B. T. *et al.* The organization of the human cerebral cortex estimated by intrinsic functional connectivity. *J. Neurophysiol.* **106**, 1125–1165 (2011).

2. Ozdemir, R. A. *et al.* Individualized perturbation of the human connectome reveals reproducible biomarkers of network dynamics relevant to cognition. *Proc. Natl. Acad. Sci. U. S. A.* **117**, 8115–8125 (2020).

3. Jenkinson, M., Beckmann, C. F., Behrens, T. E. J., Woolrich, M. W. & Smith, S. M. NeuroImage. **62**, 782–790 (2012).

4. Schaefer, A. *et al.* Local-Global Parcellation of the Human Cerebral Cortex from Intrinsic Functional Connectivity MRI. *Cereb. Cortex* **28**, 3095–3114 (2018).

| **Table S1. Detailed results of the four regression models employed.** The three values for each predictor report the regression estimates, standard errors in parenthesis and the t-statistic. | | | | |
| --- | --- | --- | --- | --- |
|  | *Dependent variable:* | | | |
|  |  | | | |
|  | Mental Health During Lock-down | | | |
|  | (Full DLPFC) | (Reduced DLPFC) | (Full IPL) | (Reduced+Baseline EEG) |
|  | | | | |
| DLPFC Local | 0.001^***^ | 0.0004^***^ |  | 0.0004^***^ |
|  | (0.0002) | (0.0001) |  | (0.0001) |
|  | t = 3.270 | t = 3.662 |  | t = 3.631 |
|  |  |  |  |  |
| DLPFC Global | -0.000 |  |  |  |
|  | (0.000) |  |  |  |
|  | t = -1.619 |  |  |  |
|  |  |  |  |  |
| IPL Local |  |  | -0.0001 |  |
|  |  |  | (0.0003) |  |
|  |  |  | t = -0.217 |  |
|  |  |  |  |  |
| IPL Global |  |  | -0.000 |  |
|  |  |  | (0.000) |  |
|  |  |  | t = -0.219 |  |
|  |  |  |  |  |
| Age | -0.013 |  | -0.011 |  |
|  | (0.009) |  | (0.013) |  |
|  | t = -1.420 |  | t = -0.823 |  |
|  |  |  |  |  |
| Gender | 0.163 |  | -0.067 |  |
|  | (0.147) |  | (0.201) |  |
|  | t = 1.107 |  | t = -0.334 |  |
|  |  |  |  |  |
| Education | -0.049^***^ | -0.053^***^ | -0.028 | -0.053^***^ |
|  | (0.017) | (0.016) | (0.025) | (0.016) |
|  | t = -2.863 | t = -3.404 | t = -1.109 | t = -3.361 |
|  |  |  |  |  |
| TMS Date | 0.013 |  | -0.026 |  |
|  | (0.020) |  | (0.024) |  |
|  | t = 0.674 |  | t = -1.105 |  |
|  |  |  |  |  |
| DLPFC Local : Targeting Method | -0.0003 |  |  |  |
|  | (0.0002) |  |  |  |
|  | t = -1.349 |  |  |  |
|  |  |  |  |  |
| DLPFC Global : Targeting Method | 0.000 |  |  |  |
|  | (0.000) |  |  |  |
|  | t = 0.976 |  |  |  |
|  |  |  |  |  |
| IPL Local : Targeting Method |  |  | 0.0001 |  |
|  |  |  | (0.0004) |  |
|  |  |  | t = 0.307 |  |
|  |  |  |  |  |
| IPL Global : Targeting Method |  |  | 0.000 |  |
|  |  |  | (0.000) |  |
|  |  |  | t = 0.281 |  |
|  |  |  |  |  |
| DLPFC Local pre-TMS Baseline |  |  |  | 0.003 |
|  |  |  |  | (0.004) |
|  |  |  |  | t = 0.668 |
|  |  |  |  |  |
| Constant | 1.798^***^ | 1.219^***^ | 2.254^**^ | 0.221 |
|  | (0.614) | (0.285) | (1.003) | (1.522) |
|  | t = 2.926 | t = 4.279 | t = 2.247 | t = 0.145 |
|  |  |  |  |  |
|  | | | | |
| Observations | 56 | 56 | 55 | 56 |
| R^2^ | 0.345 | 0.284 | 0.065 | 0.291 |
| Adjusted R^2^ | 0.234 | 0.257 | -0.097 | 0.250 |
| Residual Std. Error | 0.459 (df = 47) | 0.452 (df = 53) | 0.643 (df = 46) | 0.454 (df = 52) |
| F Statistic | 3.099^***^ (df = 8; 47) | 10.533^***^ (df = 2; 53) | 0.400 (df = 8; 46) | 7.098^***^ (df = 3; 52) |
|  | | | | |
| *Note:* | ^*^p< .05; ^**^p< .01; ^***^p< .001 | | | |
